# Development and Evolution of the Muscles of the Pelvic Fin

**DOI:** 10.1371/journal.pbio.1001168

**Published:** 2011-10-04

**Authors:** Nicholas J. Cole, Thomas E. Hall, Emily K. Don, Silke Berger, Catherine A. Boisvert, Christine Neyt, Rolf Ericsson, Jean Joss, David B. Gurevich, Peter D. Currie

**Affiliations:** 1 Victor Chang Cardiac Research Institute, Sydney, New South Wales, Australia; 2 Anatomy & Histology, School of Medical Science and Bosch Institute, The University of Sydney, New South Wales, Australia; 3 Australian Regenerative Medicine Institute, Monash University, Clayton, Australia; 4 Expression Genomics Laboratory, Institute for Molecular Bioscience, The University of Queensland, St. Lucia, Queensland, Australia; 5 Department of Biological Sciences, Macquarie University, Sydney, New South Wales, Australia; King's College London, United Kingdom

## Abstract

Locomotor strategies in terrestrial tetrapods have evolved from the utilisation of sinusoidal contractions of axial musculature, evident in ancestral fish species, to the reliance on powerful and complex limb muscles to provide propulsive force. Within tetrapods, a hindlimb-dominant locomotor strategy predominates, and its evolution is considered critical for the evident success of the tetrapod transition onto land. Here, we determine the developmental mechanisms of pelvic fin muscle formation in living fish species at critical points within the vertebrate phylogeny and reveal a stepwise modification from a primitive to a more derived mode of pelvic fin muscle formation. A distinct process generates pelvic fin muscle in bony fishes that incorporates both primitive and derived characteristics of vertebrate appendicular muscle formation. We propose that the adoption of the fully derived mode of hindlimb muscle formation from this bimodal character state is an evolutionary innovation that was critical to the success of the tetrapod transition.

## Author Summary

The transition of vertebrates from water to land is a fundamental step in the evolution of terrestrial life. Innovations that were critical to this transition were the evolution of a weight bearing pelvis, hindlimbs and their associated musculature, and the development of the “rear wheel drive” strategy that predominates in terrestrial locomotion. The fossil record can reveal how the skeletal framework of the load-bearing limbs of tetrapods (animals descended from fish) has evolved, but as soft tissues are rarely preserved within the fossil record, it can shed little light on how the accompanying dramatic alterations of the limb musculature arose developmentally. To examine this question we determined the mechanisms that generate fin muscles within larvae of living species representing several clades of fish across the vertebrate phylogeny. Using this comparative approach and a novel somite transplantation technique in zebrafish, we determine that the pelvic fin muscles of bony fish are generated by a bimodal mechanism that has features of limb/fin muscle formation in tetrapods and primitive cartilaginous fish. Using these data, we propose a unifying evolutionary hypothesis on the origins of the muscle of the paired fins and limbs, and speculate that the adoption of tetrapod mode of hindlimb muscle formation was also an evolutionary innovation critical to the success of the tetrapod transition.

## Introduction

**Figure 1:**
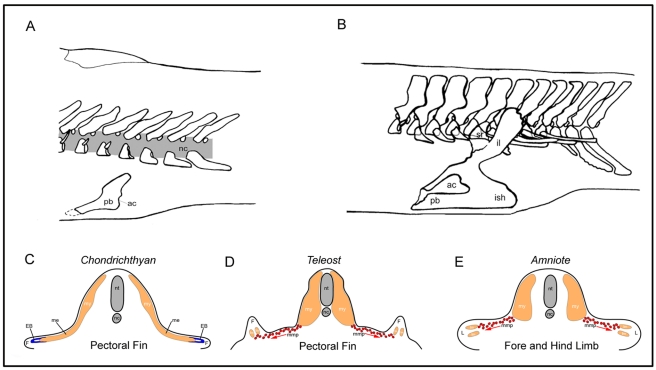
Evolution of the tetrapod pelvis and the known mechanisms of vertebrate appendicular muscle formation. (A) In sarcopterygian fish such as the extinct *Eusthenopteron*, the pelvic girdle is only supported by the hypaxial musculature and consists of a pubis (pb) with a caudally oriented acetabulum (ac) (articulation to the fin) (redrawn from [46]). (B) By contrast in early tetrapods such as *Acanthostega*, the pelvic girdle consists of a pubis, an ischium (ish), and an ilium (il), which connects to the vertebral column through the sacral rib (sr). The acetabulum is placed laterally. (redrawn from [47]) (C) Chondrichthyans utilise the primitive mechanism of direct epithelial extension to generate the muscle of the pectoral fin. (D) Zebrafish utilise the long range migration of individual mesenchymal migratory myoblasts to make the muscle of the pectoral fin. (E) Amniote limb muscle formation also occurs by the long range migration of individual mesenchymal migratory myoblasts in both the fore and hind limbs. nt, neural tube; nc, notochord; mmp, migrating muscle precursors; L, limb; lm, limb muscle; my, myotome; F, fin; ME, myotomal extension; EB, epithelial bud.

Studies of a number of fossil forms have provided information on the evolution of the appendicular skeleton of the hindlimbs within early tetrapods [1]–9. These analyses have revealed that the tetrapod transition is characterised by the gradual replacement of a relatively gracile, ventrally located pelvic girdle and laterally positioned fin endoskeleton, with a robust, dorsally positioned, pelvis and hindlimb skeleton. In tetrapods, the pelvis articulates directly with the axial skeleton via the ilium, which extends dorsally to attach to the sacral vertebrae (Figure 1A,B) [1]–[13]. Evolution of the pelvic girdle and hindlimb endoskeleton is associated with critical functional innovations within transitional tetrapods. These include additional structural support through the articulation of the pelvic girdle with the axial skeleton, increased surface area for muscle attachments, and a more lateral and dorsal positioning of the limb articulations with the axial skeleton. These adaptations are all considered essential for the evolution of load-bearing and locomotor-predominating hindlimbs.

The fossil record has, in part, charted the evolution of the skeletal framework of the load-bearing limbs of tetrapods [1]–[13]. However, individual fossils can shed little light on how the dramatic alterations of the limb musculature, required to drive locomotion in terrestrial tetrapods, arose as soft tissues are rarely preserved within the fossil record. In order to examine this question it then becomes necessary to uncover the mechanisms that generate limb and fin muscles within extant species present at crucial nodes within the vertebrate phylogeny.

Extensive analyses have been undertaken on the formation of limb muscles within two extant amniote tetrapod species, chick and mouse [14]. Both the fore and hindlimb musculature of chick and mouse embryos are generated via an identical process, in which limb myoblasts are derived from the migration of mesenchymal precursor cells. These precursors de-epithelialise from the ventro-lateral, or hypaxial, region of limb-level somites and undergo a long range migration to their final position within the limb mesenchyme (Figure 1E) [15]. During this process, these cells require the expression of a number of specific genes including the homeobox-containing gene *Lbx*.

*Lbx* expression is important in the context of this study as in amniotes it is a highly specific marker of migratory muscle precursor cells within the limb-adjacent somitic mesoderm and is also maintained within migrating and post-migratory limb myoblasts. *Lbx* is not only expressed within amniote limb myoblasts but is also functionally required for their formation and correct differentiation. Homozygous *Lbx* mutant mice fail to form limb muscle normally with extensor muscles of the forelimbs being absent and flexor muscles reduced in size. Hindlimb muscles are also strongly affected, with distal limb muscles more affected than proximal ones [14],[16]–[18].

A similar, *lbx1*-positive, set of fin muscle precursors—also derived from the migration of mesenchymal precursor cells that originate from pectoral fin level somites [19]—have been shown to generate the appendicular muscle present within the pectoral fin of the teleost, zebrafish (Figure 1D). Thus, the pectoral fin muscle precursors of zebrafish possess molecular and morphogenetic identity to the limb muscle precursors of tetrapod species [19]–[21]. However, how widely within the bony fish phylogeny this mechanism is deployed has yet to be determined.

In contrast, we have previously shown that the embryos of the shark *Scyliorhinus canicula* (a chondrichthyan species basally positioned in the vertebrate phylogeny) utilise a separate process of direct epithelial extension from the embryonic myotome to generate both the hypaxial muscles of the body wall, and secondarily at its most ventral extent, the muscles of the pectoral fins. This process is characterised by the progressive extension of the myotome, via a ventrally displacing epithelial bud, that directly enters the fin to generate the muscle of the pectoral fin without the migration of *lbx1* expressing mesenchymal precursors (Figure 1C). Given the basal position of chondricthyans within the vertebrate phylogeny we have defined the mechanism of direct epithelial myotomal extension as the primitive mode of appendicular muscle formation. In this paradigm, the generation of limb myoblasts in amniotes and pectoral fin muscle in zebrafish via *lbx*-positive migratory mesenchymal precursor myoblasts represents the derived mode of appendicular muscle formation [19].

By contrast, the developmental origin and molecular processes that generate pelvic fin muscle have not been defined in any fish species to date. To understand the changes underlying the evolution of the pelvic fin musculature, we have studied the mechanisms of pelvic fin muscle formation in living fish species positioned at strategic points within the vertebrate phylogeny. Here we reveal that all bony fish species we have examined make pelvic fin muscle using the same developmental process, utilising a myotomal extension to deliver fin muscle precursors adjacent to the forming pelvic fin. Once in position adjacent to the pelvic fin bud, muscle precursors undergo an epithelial mesenchymal transition and are induced to express *lbx1* and migrate into the fin mesenchyme to form individual pelvic fin muscles. Collectively, these studies demonstrate that the pelvic fin musculature of bony fish is generated by a novel morphogenetic process that possesses characteristics of both the primitive (epithelial myotomal extension) and derived (lbx1-positive migratory mesenchymal myoblast precursors) modes of vertebrate appendicular muscle formation. We further propose that the adoption of the fully derived mode of hindlimb muscle formation from this bimodal character state was an evolutionary innovation critical to the success of the tetrapod transition.

## Results

### Pectoral and Pelvic Fin Muscle Formation in Fish Species Positioned at Critical Junctures in the Vertebrate Phylogeny

We have compared the mechanism of fin muscle development of two chondrichthyan cartilaginous fish species (bamboo shark, *Chiloscyllium punctatum* and the chimera, *Callorhinchus milii*) and three bony fishes, the North American paddlefish, *Polyodon spathula*, a teleost (zebrafish *Danio rerio*), and the Australian Lungfish *Neoceratodus forsteri*.

The bamboo shark, *Chiloscyllium punctatum* and the chimera *Callorhinchus milii* are basal in the vertebrate phylogeny. *C. milii* as a Chimaeriform is considered basal within the chondricthyan clade, with the callorhynchids representing the most primitive living members of the Holocephali [22]. Thus, *C. milii* is a representative of the most primitive extant fish species with paired appendages. Thus, the common developmental features shared by the shark and chimera are expected to represent the primitive state of fin/limbed vertebrates.

Of the bony fishes examined, the North American paddlefish, *Polyodon spathula*, is a living representative of a group of primitive Chondrostean ray-finned (actinopterygian) fish and occupies an important basal position within the bony fish. The Australian lungfish, *Neoceratodus forsteri*, is the only example of a lobe fin (sarcopterygian) fish for which embryonic material can be obtained, and as such is a critically positioned species for understanding the evolution of the tetrapod transition. Finally, the zebrafish family (*Cyprinidae* of the order *Cypriniformes*) represents a teleost and is a genetically tractable established model vertebrate that is amenable to a myriad of powerful molecular techniques allowing greater resolution/depth to the precise nature of the developmental mechanisms occurring.

We focused our analysis on the periods of development of these distinct species when the fins are initially formed. We utilised the presence of epithelial buds at the head of myotomal extensions within the fin mesenchyme as an indicator of the primitive mode of fin muscle formation [19]. Conversely, the lack of an epithelial myotomal extension and the expression of *lbx1* within mesenchymal migratory fin myoblasts were used as markers for the derived mode of fin muscle precursor migration [19].

**Figure 2:**
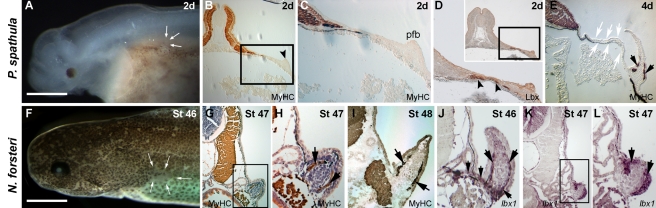
Pectoral fin muscle formation in paddlefish (*Polyodon spathula*) and lungfish (*Neoceratodus forsteri*) utilises the fully derived mode of appendicular muscle formation and is not associated with an epithelial extension. (A–E) Pectoral fin muscle formation in paddlefish (*Polyodon spathula*). (A) At 2 d post-hatching (dph) the pectoral fin is present as a bud (arrows). Scale bar 1 mm. (B) A 2 dph embryo transverse section stained for MHC (brown). The pectoral fin is initially present as a fin bud (arrowhead). (C) Magnification of the region boxed in (B) (pfb, pelvic fin bud). (D) Immunohistochemmistry of a section, serial to that in (C), with an anti-Lbx antibody reveals Lbx-positive fin myoblasts migrating as small groups of mesenchymal cells towards the pectoral fin. (E) At 4 dph the larvae possess differentiated muscle evident within the pectoral fins, and a gap (arrows) with no differentiated muscle between the fin and the myotome as the muscle precursors have migrated into the fin bud prior to differentiation (pf, pectoral fin). (F–L) Pectoral fin muscle formation in lungfish (*Neoceratodus forsteri*). (F) At stage 46 the pectoral fin is present as a bud (arrows). Pelvic fin buds are not yet present at this stage. Scale bar 1 mm. (G) At stage 47 pectoral fin muscle differentiation, stained for MHC (brown), is discrete with the fin with no evidence of a myotomal extension. (H) High magnification view of the region boxed in (G). Counterstain (H&E). (I) Stage 48 lungfish stained for MHC alone reveals the formation of the dorsal and ventral muscle masses of the pectoral fin, without an association of a myotmal extension. (J) Migratory *lbx1*-positive cells (purple, indicated with arrows) are present both between the developing pectoral fin and myotome and within the fin at stage 46. No myotomal extension is evident. (K) *lbx1*-positive cells (purple) are present within fin at stage 47. No myotomal extension is evident. Section level is at the anterior base of the pectoral fin. Boxed region magnified in (L) (*lbx1*-positive cells within the pectoral fin muscle masses are purple, indicated with arrows).

Our initial studies focused on confirming the deployment of the derived mode of appendicular muscle formation in the pectoral fin of bony fish other than zebrafish. We undertook this analysis in order to strengthen the phylogenetic assignment of this character state as having arisen prior to the sarcopterygian radiation, as a mosaic distribution of this mode of appendicular muscle formation has been previously reported to occur within the bony fish phylogeny (extensively reviewed in [23], see below). Within both paddlefish and lungfish embryos there was no evidence of a myotomal extension, and *lbx*-positive pectoral fin muscles were generated discretely within the fin bud (Figure 2, unpublished data). In embryos of these species muscle differentiation occurred within the pectoral fin and generated defined dorsal and ventral fin muscle masses, without any connection to an epithelial somitic extension (Figure 2B). This analysis confirmed the presence of the derived mode of appendicular muscle formation in the pectoral fin of paddlefish and lungfish analogous to that seen in our previous studies on zebrafish pectoral fin muscle development [19].

**Figure 3:**
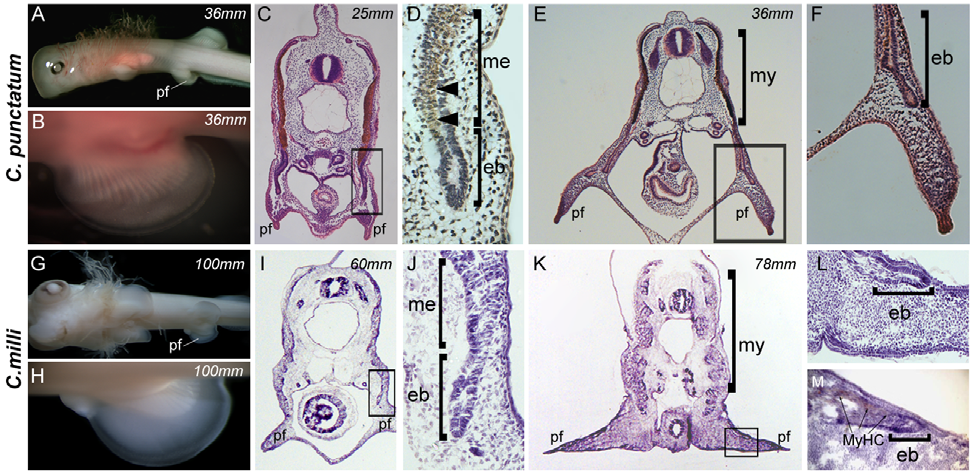
Pelvic fin muscle formation in the chondricthyans. Pelvic fin muscle formation in *Chiloscyllium punctatum* (bamboo shark) (A–F) and *Callorhinchus milii* (Chimera) (G–L). (my, myotomes; nt, neural tube; nc, notochord; l, limb; mmp, migrating muscle pioneers; f, fin; ep, epithelial bud; me, myotome extension; pf, pelvic fin). Arrowheads in (D) and arrows in (M) denote differentiating muscle fibres detected with an antibody to Myosin Heavy Chain (Brown). Total length of the specimens is noted in mm.

We next turned our attention to the mechanisms that generate pelvic fin muscle formation. As mentioned above, the morphogenetic and molecular basis for pelvic fin muscle development has not been determined for any fish species to date. We first examined pelvic fin muscle formation in the two chondricthyan species under study. Within both species, the pelvic fin muscles were generated by direct epithelial extension of the myotome, headed by a characteristic migrating epithelial bud (Figure 3A–M). Epithelial buds progressively generate the muscle of the body wall as they extend ventrally from the myotome and, at their most ventral extent, enter the forming pelvic fin mesenchyme to generate the pelvic fin muscles (Figure 3A–M). Furthermore, although lbx1 expression could be detected by antibody labelling within the neural tube of *C. milli*, a known site of lbx1 expression in zebrafish embryos [19], no expression could be detected within its fin epithelial myotomal extensions (Figure S4). Collectively, the above data strongly suggest that the pelvic fin muscle of chondrichthyan species is generated by the primitive mode of direct epithelial extension of fin-adjacent myotomes, in a process identical to that described to generate the muscles of the chondrichthyan pectoral fin.

**Figure 4:**
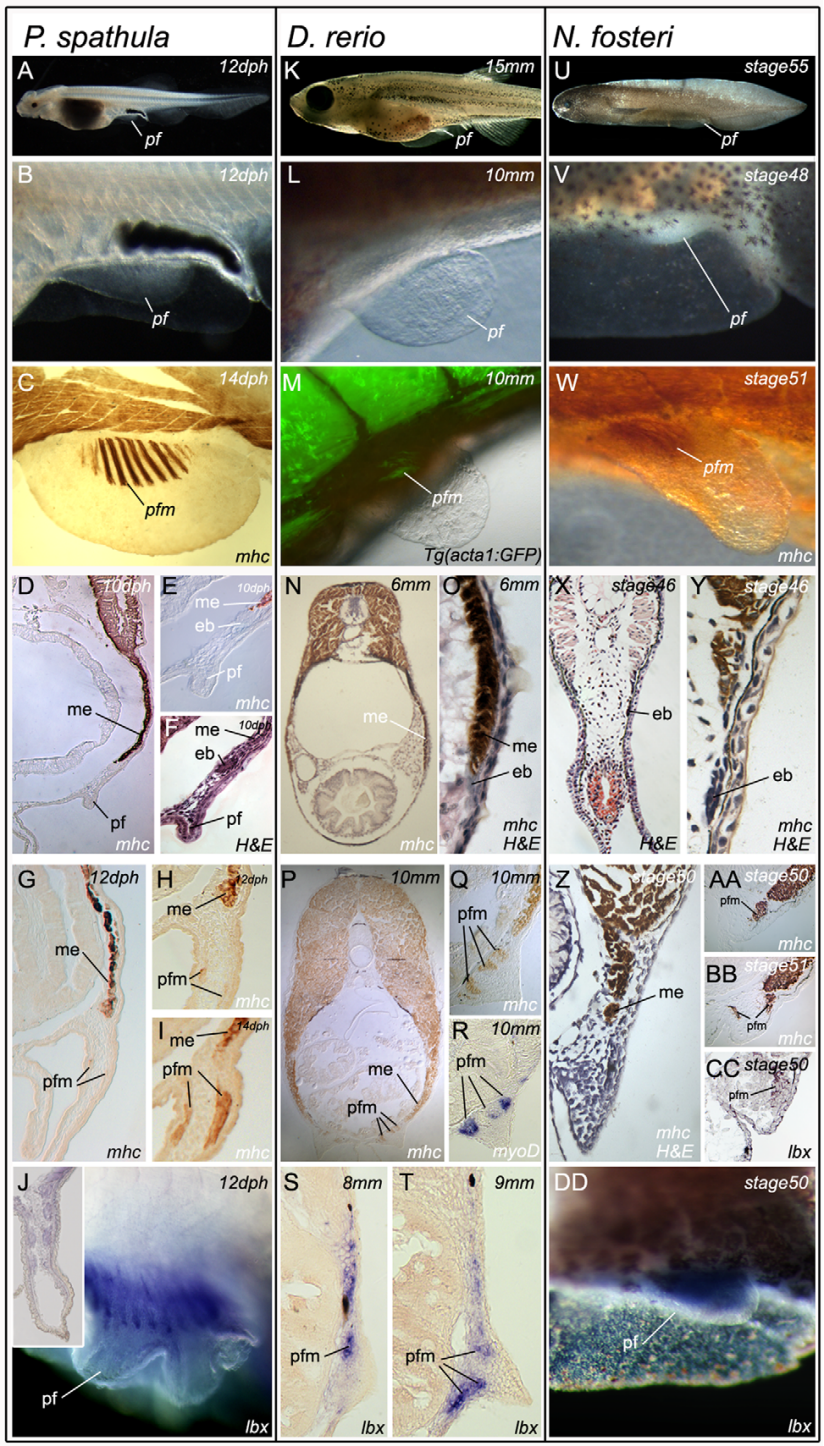
Pelvic fin muscle formation in bony fish. Pelvic fin muscle formation in *P. spathula* (A–J), *D. rerio* (K–T), and *N. forsteri* (U–DD). Larvae at stages when pelvic fin muscles form (A, K, and U). Developing pelvic fin bud (B, L, and V). Muscle fibres in developing pelvic fin are separate and distinct from the muscle of the somite (C, M, and W). Immediately before pelvic fin formation epithelial buds (mb) head the myotomal extension (me) (D, E, F, N, O, X, Y). The pelvic fin muscles (pfm) have formed within the pelvic fin and are separate from the myotomal extension (me) (G, H, I, P, Q, Z, AA, BB). *myoD* is restricted to individual, post-migratory, differentiating pelvic fin muscles (R). *lbx1* positive cells (purple) at the position of the forming pelvic fin muscles (pfm) in lungfish (DD). *Lbx1* expression in 14 dph pelvic fin of *P. spathula*, inset is a cross-section of the pelvic fin at the same stage, revealing *lbx* expression in the muscle masses (J). *Lbx1* positive (blue) precursors in the tip of the extension position of the future pelvic fin muscles in *D. rerio* at 8 mm TL (S) and 9 mm TL (T). *Lbx1*-positive precursors in stage 50 pelvic fin bud of *N. forsteri* (DD). (ep, epithelial bud; me, myotome extension; pf, pelvic fin; pfm, pelvic fin muscle).

Similarly, in all three bony fishes examined, myosin heavy chain (MHC) positive cells were detected extending ventrally from the somite towards the future position of the developing pelvic fins (Figure 4D,E,N,O,X,Y). In each case the extension was headed by an epithelial bud that progressively generated the hypaxial muscle of the body wall as it extended towards the level of the pelvic fin bud. However, in contrast to chondricthyan embryos, the myotomal extension of all bony fish examined failed to enter the fin bud mesenchyme. This was despite having arrived at its most ventral extent temporally and spatially coincident with the formation of the adjacent pelvic fin bud (Figure 4E–I,N–Q,X–BB). Furthermore, the first differentiated muscle cells that appeared within the pelvic fins of each species were clearly separate from the myotomal extension, which by this stage lacked any evidence of an epithelial character (Figure 4H,I,P–R,Z–BB). Muscle differentiated within the fin of each species with no evidence of the myotomal extension entering the forming pelvic fin bud (Figure 4G–I,P–R,Z–BB).

In contrast to the epithelial extension evident during chondrichthyan paired fin muscle formation, in situ hybridization revealed that *lbx1* mRNA is expressed in the tip of the fin adjacent to myotomal extension, and in fin muscle precursors during their short range migration from the extension into the pelvic fin (Figure 4J,S,T,DD, Figure S2). This migration was most clearly seen in the zebrafish by section in situ hybridization where *lbx1*-positive mesenchymal cells migrated towards the developing fin bud mesenchyme such that at 9 mm TL, two distinct regions of *lbx1* expression were evident within the pelvic fin, corresponding to the future dorsal and ventral muscle masses of the pelvic fin (Figure 4T).

Taken as a whole, these data suggest that a similar mechanism to that operating in zebrafish generates paddlefish and lungfish pelvic fin musculature. This mechanism is a bimodal character state comprising features of both the primitive and derived modes of vertebrate appendicular muscle formation.

### Somite Transplantation Defines the Developmental Origin of Pelvic Fin Muscle Precursors in Zebrafish

**Figure 5:**
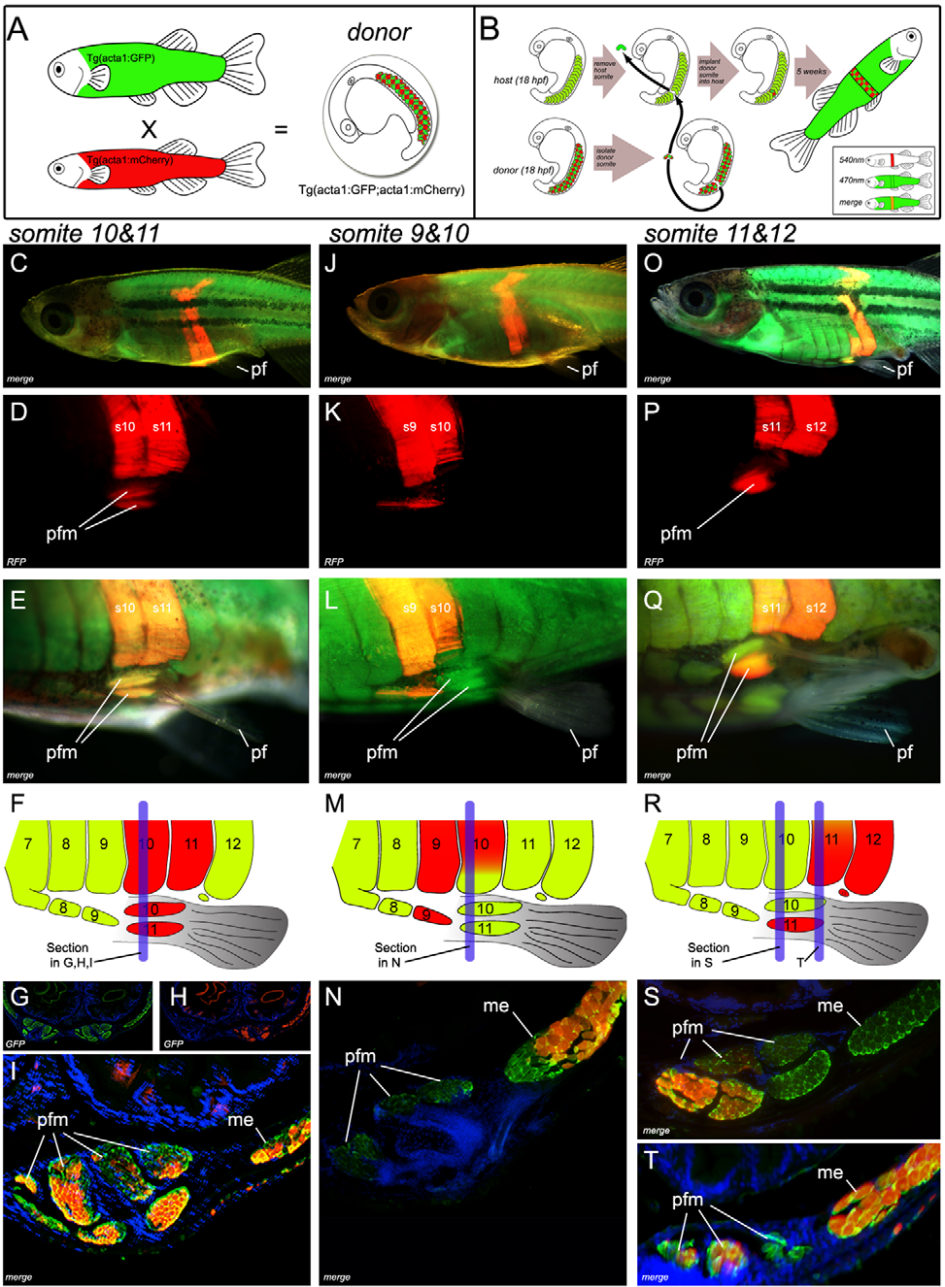
Transgenic somite transplantation in *D. rerio* reveals pelvic fin muscles derive from myotomal extension. (A) Donor embryos are generated by crossing *Tg(acta1∶mCherry)^pc4^* with *Tg(acta1∶GFP)^zf13^* [33]. (B) Donor somites are surgically removed and transplanted into the host. (C–I) Myotomal extensions derived from somites 10 and 11 generate the pelvic fin muscles (Section through s10 (blue line in F) of host (5 wk post-operation) (G, H, and I)). (Comprehensive methods are provided in supplementary data, however surgery involving transplantation of two consecutive somites lead to a greater probability of transplanting the entire somite, including the ventral aspect required for pelvic fin muscle formation). (J–L) Contribution to the pelvic fin muscle requires the most ventral tip of the somite to be transplanted. Full transplantation of somite 9 contributes to ventral muscle anterior to pelvic fin. but a partial transplant of somite 10 does not result in contribution to the pelvic fin muscle. (M, N) Section through s10 (blue line in M) of the transplanted host (5 wk post-operation). (N) If the donor somite is not included in the most ventral tip of the extension, contribution to the fin does not occur . (O–T) The most ventral group of pelvic fin muscles are derived from somite 11 (s11+s12) section through s10 (S) (blue line in R) and s11 (T) (blue line in R) of host (5 wk post-operation). Pf, pelvic fin; rfp, red fluorescent protein; pfm, pelvic fin muscle; me, myotomal extension; s9,s10,s11,s12, somite numbered from anterior to posterior.

Although these morphological and gene expression studies suggest the origin of the pelvic fin muscle lies within the adjacent epithelial myotomal extensions, they do not provide direct evidence for it. The heterochronous development displayed by the pelvic fins of zebrafish (a primitive character of vertebrates with paired appendages shared by most fish [24],[25]), which develop 4 wk after formation of the pectoral fin bud initiates at the end of somitogenesis has precluded an examination of the developmental origin of the cells of the pelvic fin musculature, as no fate mapping strategies have been developed in fish that allow tracking of somite derived cells for this period of time. Somite transplantation has been deployed in other model systems, most successfully by chick embryologists, where it has been used to determine the fates of somitic cells. Historically, the technique was utilized to determine somitic origin of limb myoblasts, a finding that overturned the prevailing hypothesis that limb myoblasts originated from lateral plate mesoderm [15]. In the context of this current analysis somite transplantation has the advantage that genetically distinct donor tissue is indelibly marked and can be used to determine donor tissue contribution to a host structure at any developmental time point. Thus, we developed a transgenic fate mapping strategy that enabled the transplantation of embryonic somitic tissue between different genetically marked strains of zebrafish. In this strategy donor embryos are generated by crossing adults transgenic for mCherry driven by the muscle specific alpha actin promoter *Tg(acta1∶mCherry)^pc4^* with those carrying a transgene that drives GFP off the same promoter *Tg(acta1∶GFP)^zf13^* to generate donor embryos marked both with red and green fluorescence in the nascent myotome. This strategy was necessary because mCherry gives a weaker signal and consequently was not highly visible at the embryonic stages at which transplantation can be carried out. Thus, in order to enable specific dissection of somitic tissue, the brighter GFP construct was crossed into the background of the donor. Isolated donor somites were then transplanted homotypically into a “green only” *Tg(acta1∶GFP)^zf13^* host in which the host level somite had been extirpated. Transplanted embryos were grown to adulthood and donor tissue contribution to the host assessed via mCherry fluorescence. Using this strategy we were able to observe *in vivo*, and in real time, the fate of the transplanted somitic cells through the entire life span of the fish (Figure 5A,B, Materials and Methods).

Under this transplant regime, mCherry positive donor somites formed the ventral somitic extension, which generated the body wall musculature at all transplant levels. In order to examine if any cell type other than muscle was generated by the ventral somitic extension, a triple transgenic fluorescent transplant strategy was developed. In this strategy, a ubiquitously expressed promoter (beta actin) drives mCherry in the donor somite, which is also marked with GFP driven by the alpha actin skeletal muscle-specific promoter. The donor somite is transplanted into a host that is transgenic for BFP also expressed via alpha actin skeletal muscle-specific promoter. In each of 12 transplants performed in this way, only co-expression of both green and red fluorescent protein was ever observed, indicating that the donor somite contained only somitic tissue and that this only ever generated donor-derived muscle in the host (Figure S1).

At the level of the pelvic fin, the extension contributed to the pelvic fin muscle on the operated side, with individual somites transplanted at specific somitic levels giving rise to specific muscles within the pelvic fin (*n* = 6; Figure 5A–T). By contrast, the non-operated (contralateral) side never showed mCherry-positive cells within the pelvic fin muscle masses. Furthermore, somite transplantation anterior (*n* = 6) and posterior (*n* = 4) to somites 10 and 11 did not reveal any contribution to the pelvic fin muscles (Figure S3). This procedure revealed that the pelvic fin muscles of zebrafish originate from pelvic fin level somites. Furthermore, in order for a donor somite to contribute to the pelvic fin muscles, the donor tissue has to be present in the most ventral portion of the extension, as transplants where host tissue remained at the most ventral tip ahead of the donor tissue resulted in only host tissue contributing to the pelvic fin muscle (Figure 5J–N).

These data therefore illustrate that fin muscle precursors of zebrafish are contained within, and are carried ventrally by, myotomal extension. Once in position adjacent to the pelvic fin bud, muscle precursors undergo an epithelial mesenchymal transition and are induced to express *lbx1* and migrate into the fin mesenchyme to form individual pelvic fin muscles. Collectively, these studies demonstrate that the pelvic fin musculature of bony fish is generated by a novel morphogenetic process that possesses characteristics of both the primitive (epithelial myotomal extension) and derived (*lbx*1-positive migratory mesenchymal myoblast precursors) modes of vertebrate appendicular muscle formation.

## Discussion

Through these studies we have morphologically and molecularly defined a developmental mode of fin muscle formation that is intermediate between the primitive mechanism of paired fin muscle formation, evident in chondrichthyan species, and the derived mode, evident in the pectoral fin of bony fishes and the fore and hindlimbs of tetrapods [19]. The existence of such a bimodal character state has been postulated previously, but evidence as to its existence as well as an understanding of its evolutionary significance have both been lacking [23],[26],[27].

**Figure 6:**
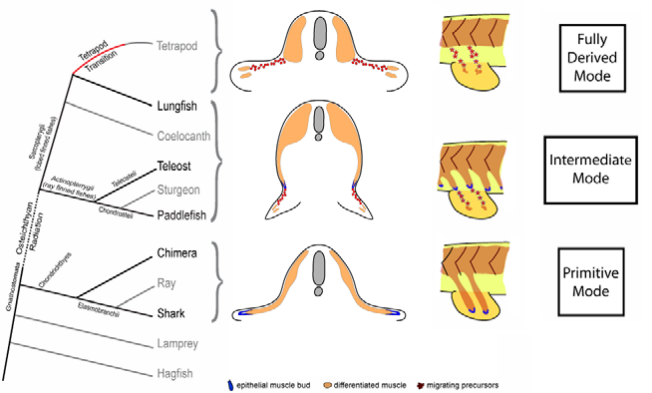
A phylogenetic framework for pelvic fin muscle evolution. Evolution of pelvic fin and hindlimb developmental mechanisms. Pelvic fin and hindlimb developmental mechanisms mapped onto the vertebrate phylogeny.

Our observations also resolve the previously reported mosaic distribution of the primitive and derived modes of appendicular muscle formation [15],[19],[23],[26]–[34] into a single phylogenetically harmonious framework (Figure 6). Indeed, we think it likely that the existence of this bimodal character state was difficult to resolve with only the tools of simple histology available to the researchers at the time that many of these studies were performed. The existence of an epithelial extension associated with pelvic fin muscle formation may well have caused confusion as to the nature of the mechanisms deployed in both sets of paired fins.

In order to locomote on land, the robust, dorsally articulated, pelvic girdle and expanded hindlimb skeletons present in tetrapod species need to be populated by powerful hindlimb muscles that not only support the weight of the whole animal but also coordinate movement. Critically, we believe, removing the requirement for pelvic fin muscle formation to be coupled to the ventral arrival of a myotomal extension provided flexibility in pelvic fin/limb positioning without compromising body wall muscle formation. The possible structural limitations arising from the deployment of myotomal extension are evident if we consider that it is used to generate all hypaxial muscles of the body wall by continuous ventral extension of the somitic epithelial bud. Upon the arrival of the somitic bud at is most ventral extent, the bud undergoes an epithelial to mesenchymal transition to generate the muscles of the pelvic fins. Thus, use of this mode of muscle formation precludes hypaxial muscle formation ventral to the position of the pelvic fin, as continued ventral formation of hypaxial muscle cannot occur in the absence of the somitic bud. In support of this hypothesis a lack of hypaxial muscle formation ventral to the pelvic fin is evident in all the species we examined (Figure 4). Thus, upon the adoption of the fully derived mode of appendicular muscle formation the pelvic fin was released from the constraint of having to be positioned ventral to the hypaxial muscle of the body wall. Consequently, the pelvic fin could be located anywhere in the dorsoventral body axis (a plasticity already evident in the positioning of the pectoral fin of bony fishes), facilitating the dorsal shift in pelvic girdle location necessary for direct articulation with the axial skeleton and the development of load-bearing hind limbs. It would also allow the pectoral and pelvic fins to develop relatively synchronously, a process characteristic of the fore and hind limbs of model tetrapod species, which is in contrast to the primitive condition, described for both *Chondrichthyes* and *Osteichthyes*, where the pectoral fin invariably develops prior to the formation of the pelvic fin [24],[25]. Furthermore, the ability to deploy muscle progenitors into the pelvic fin/limb environment at a relatively earlier phase of body plan development, prior to the completion of hypaxial body wall formation, may have facilitated the development of the more complex and physically larger sets of muscle required for terrestrial locomotion.

We therefore consider that the novel method of pelvic fin formation we describe in bony fish may represent an important intermediate step in the evolution of tetrapod limb muscle developmental mechanisms. We hypothesise that the adoption of the fully derived mode of hindlimb muscle formation was an evolutionary innovation critical to the success of the tetrapod transition. Data in amphibian species support this notion as several studies, as well as our own unpublished observations, have failed to detect epithelial extensions associated with the formation of pelvic fin muscle in both *Amblystoma puncatum* and *Xenopus laevis* [35],[36], despite early controversy as to the presence or absence of epithelial extension in these species [37],[38]. Histological and gene expression studies have revealed that *Xenopus* hindlimb muscle precursors express markers associated with migratory limb muscle precursors of amniotes, and differentiate discretely within the limb bud, devoid of an association with an epithelial bud [39],[40]. Furthermore, recent studies have shown that *lbx* expression is associated with migratory limb muscle precursors in both fore and hind limbs of the direct developing frog *Eleutherodaclylus coqui* [41]. Collectively, these studies suggest that amphibians adopted the fully derived mode of limb muscle formation during the tetrapod transition.

## Material and Methods

### Immunochemistry and in situ Hybridisation

Whole-mount immunohistochemistry and in situ hybridization on shark, paddlefish, lungfish, and zebrafish embryos and larvae were carried out as described [19]. Cryostat, wax sections, and counterstains were carried out as described [19]. Some *C. milli* sections were obtained from museum specimens archived in ethanol and required extensive antigen retrieval to detect MHC expression. Sections were incubated in sodium citrate buffer (10 mM Sodium Citrate, 0.05% Tween 20, pH 6.0) at 95°C for 40 min, cooled for 20 min, and sections rinsed in PBS 0.05% Tween 20 for 2×2 min before incubation with antibody. Primary antibodies used were: anti-myosin 1∶200 (A4-1025, Developmental Studies Hybridoma Bank) and anti-LBX1 1∶1000 (ab90839, Abcam). Antibody binding was visualized by standard techniques [19].

### Whole Somite Transplantation

Donor (*Tg(acta1∶mCherry)^pc4^*) and host (*Tg(acta1∶GFP)^zf13^*) [42] embryos were stage matched from syncronous spawnings and transplantations undertaken at the 15 somite stage. Donor embryos were either singly transgenic for *α-actin-mCherry* or doubly transgenic for both *α-actin-mCherry* and *α-actin-GFP*. Use of the double transgenic donors greatly aided the initial dissection of donor somites, as the slow maturation rate of the mCherry produced donor somites that were only weakly fluorescent at the initial transplantation stage. Somites from the donor animal were collected on ice following dissection and pancreatin treatment in DMEM medium. Host embryos were embedded in 1% agarose with 0.016% tricaine (pH7) and submerged in DMEM medium. One or two somites were removed from the required position by dissection with flame sharpened tungsten needles. Surgery involving transplantation of two consecutive somites gave a greater probability of transplanting the entire somite, including the ventral aspect required for pelvic fin muscle formation. It also led to greater transplant survival. The donor somite(s) was then aligned and inserted into the extirpated somite region and the embryo was allowed to recover for 2 h before dissection from the agarose and rearing in E3 medium containing antibiotic (1,000 U/mL Penicillin-G 1,000 µg/mL Streptomycin) for 2 d. Fish were then reared under standard laboratory conditions for 6 wk and the transplant observed regularly under a dissecting fluorescent microscope.

### Production of Stable Transgenic Lines

The *Tg(bact2∶mCherry)^pc3^*, *Tg(acta1∶mCherry)^pc4^*, and *Tg(acta1∶EBFP2)^pc5^* transgenic lines were created using the Tol2kit [43]. The vectors used for transgenesis were assembled from appropriate combinations of the entry clones p5E-acta1 [44], p5E-bact2, pME-mCherry, pME-EBFP2, p3E-polyA, and the destination vector pDEST-tol2-pA2. We generated pME-EBFP2 by PCR subcloning from pBAD-EBFP2 [45]. The primers used for PCR amplification were: EGFP/EBFP2_F1_pME 5′- GGGGACAAGTTTGTACAAAAAAGCAGGCTggaccatggtgagcaagggcgaggagctgtt -3′ and Flouro-STOP-pME 5′- GGGGACCACTTTGTACAAGAAAGCTGGGTgttacttgtacagctcgtccatgc -3′ (Gateway sites shown in upper case).

### Identification of Lungfish Paddlefish and Bamboo Shark *lbx* Gene Homologues and *C. milii* Husbandry

We identified fragments of the lungfish, paddlefish, and bamboo shark *lbx1*genes, encoding the homeodomain, from complementary DNA pools prepared from embryos of each species by using degenerate PCR primers previously described [19]. Nucleotide and amino acid alignment of lungfish, paddlefish, bamboo shark, and zebrafish LBX to each other and human LBX proteins is included in the Supporting Information section. The lungfish, paddlefish, and bamboo shark *lbx1* fragments isolated exhibit 79%, 82%, and 82%, respectively, of sequence identity over a 182-bp region of the homeodomain. Lungfish, paddlefish, and bamboo shark *lbx1* sequences have been submitted to Genbank accession nos. EU937814, EU937815, and EU937816. Impregnated females of *Callorhinchus milii* were line caught during breeding season in Western Port Bay, Victoria, Australia. They were transferred to holding tanks for a month while they laid eggs in captivity. Eggs were labelled with the deposition date and were opened at regular intervals, staged, and fixed in the laboratory with 4% PFA using standard procedures.

## Supporting Information

Figure S1 Donor somite transplants contribute only muscle to the host. (A) Fish transgenic for a ubiquitously expressed promoter (beta actin2), expressing mCherry (*Tg(bact2∶mCherry)^pc3^*), are crossed with fish transgenic for GFP driven by the alpha actin skeletal muscle specific promoter (*Tg(acta1∶GFP)^zf13^*). (B) The recipient host embryos are transgenic for BFP also expressed via the alpha actin skeletal muscle specific promoter (*Tg(acta1∶BFP)^pc5^*). (C) The donor somites from embryos doubly transgenic for *Tg(bact2∶mCherry^pc3^)* and *Tg(acta1∶GFP^zf13^)* were transplanted into a *Tg(acta1∶BFP^pc5^*) host. In each of 12 transplants performed in this way, co-expression of both green and red fluorescent protein was only ever observed, indicating that the donor somite somitic tissue only ever generated donor-derived muscle in the host. (D–G) Transplant of somite 10 using the method outlined in (C).
(TIF)

Figure S2 Identification of *lbx* sequences in different fish species. *lbx1* nucleotide (top) and amino acid (bottom) alignments for human (*H. sapiens*), lungfish (*N. forsteri*), zebrafish (*D. rerio*), paddlefish (*P. spathula*), and bamboo shark (*C. punctatum*). Variations between the sequences are shown in grey.
(DOC)

Figure S3 Myotomal extensions derived from somite 8 generate the individual muscle adjacent to the ventral tip of the extending myotomes and anterior to pelvic fin. Pf, pelvic fin; pfm, pelvic fin muscle; rfp, red fluorescent protein; s8, the 8th somite numbered from anterior to posterior.
(TIF)

Figure S4 Lbx protein is not detected in the epithelial extension of *C. milii*. (A) Cross-section at the level of the pelvic fin (pf) of a 28 stage *C. milii* embryo incubated with an antibody against Lbx1. Expression is detected within the neural tube (nt, arrow head), a known region of expression for Lbx in other species, but is not evident within the epithelial bud (eb) of the myotomal extension. (B) Magnification of the area boxed in (A) showing the expression of Lbx in the neural tube (arrow head). (C) Lbx expression is absent from the epithelial bud of the myotomal extension. (D) Sense probe control for *lbx* in situ hybridisation on whole mounts of 12 dph paddlefish, the stage utilised in Figure 4J. (E) Sense control for *lbx* in situ hybridisation on stage 50 lungfish embryos, the stage utilised in Figure 4DD. nc, notochord.
(TIF)
