# Supplementary figures and images for: Development and Evolution of the Muscles of the Pelvic Fin
Source: PLoS Biol. 2011 Oct 4;9(10):e1001168. doi: 10.1371/journal.pbio.1001168 (PMC3186808; doi:10.1371/journal.pbio.1001168)

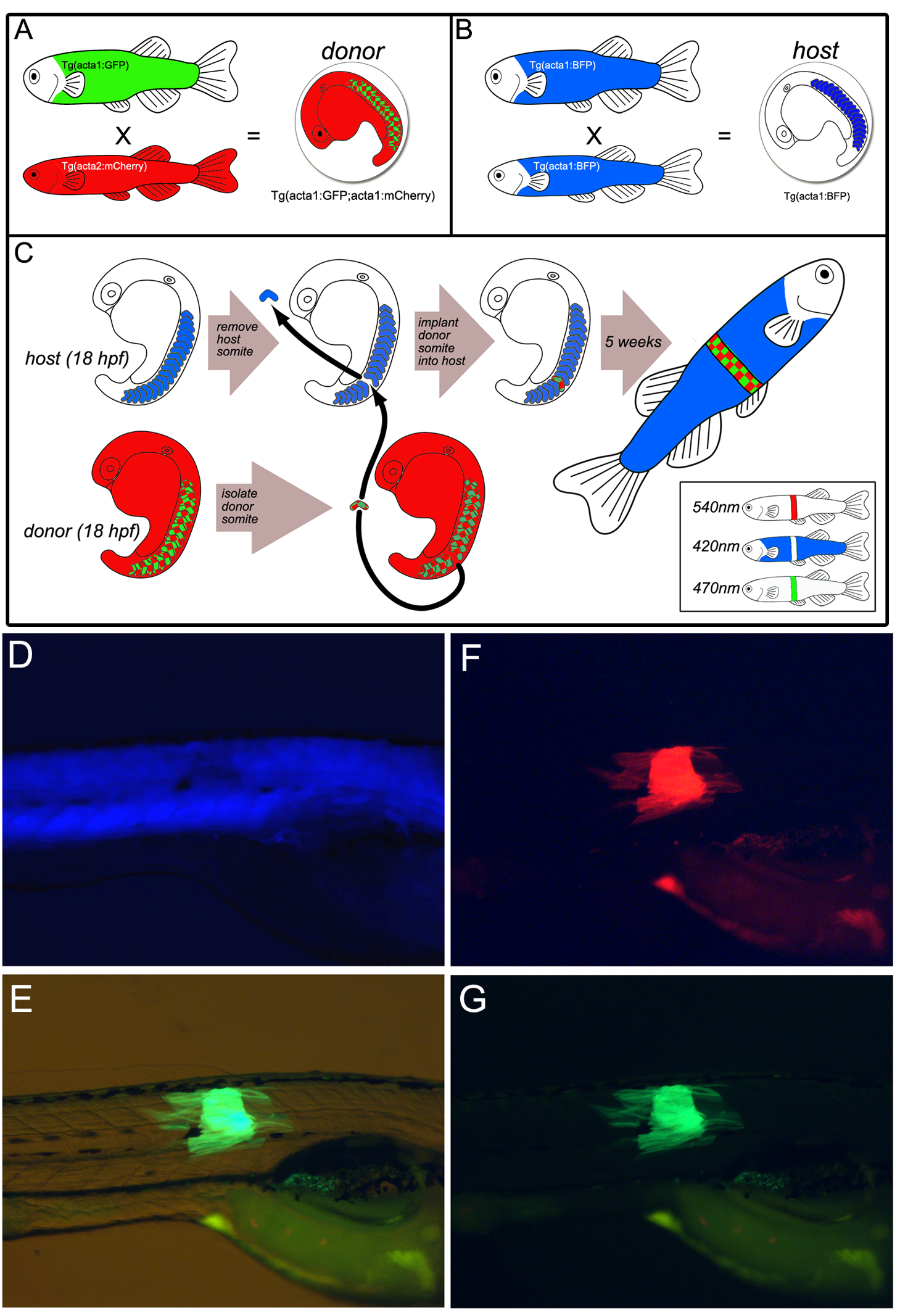

Supplement: Figure S1 — Donor somite transplants contribute only muscle to the host. (A) Fish transgenic for a ubiquitously expressed promoter (beta actin2), expressing mCherry (Tg(bact2∶mCherry)pc3), are crossed with fish transgenic for GFP driven by the alpha actin skeletal muscle specific promoter (Tg(acta1∶GFP)zf13). (B) The recipient host embryos are transgenic for BFP also expressed via the alpha actin skeletal muscle specific promoter (Tg(acta1∶BFP)pc5). (C) The donor somites from embryos doubly transgenic for Tg(bact2∶mCherrypc3) and Tg(acta1∶GFPzf13) were transplanted into a Tg(acta1∶BFPpc5) host. In each of 12 transplants performed in this way, co-expression of both green and red fluorescent protein was only ever observed, indicating that the donor somite somitic tissue only ever generated donor-derived muscle in the host. (D–G) Transplant of somite 10 using the method outlined in (C). (TIF) [file pbio.1001168.s001.tif]

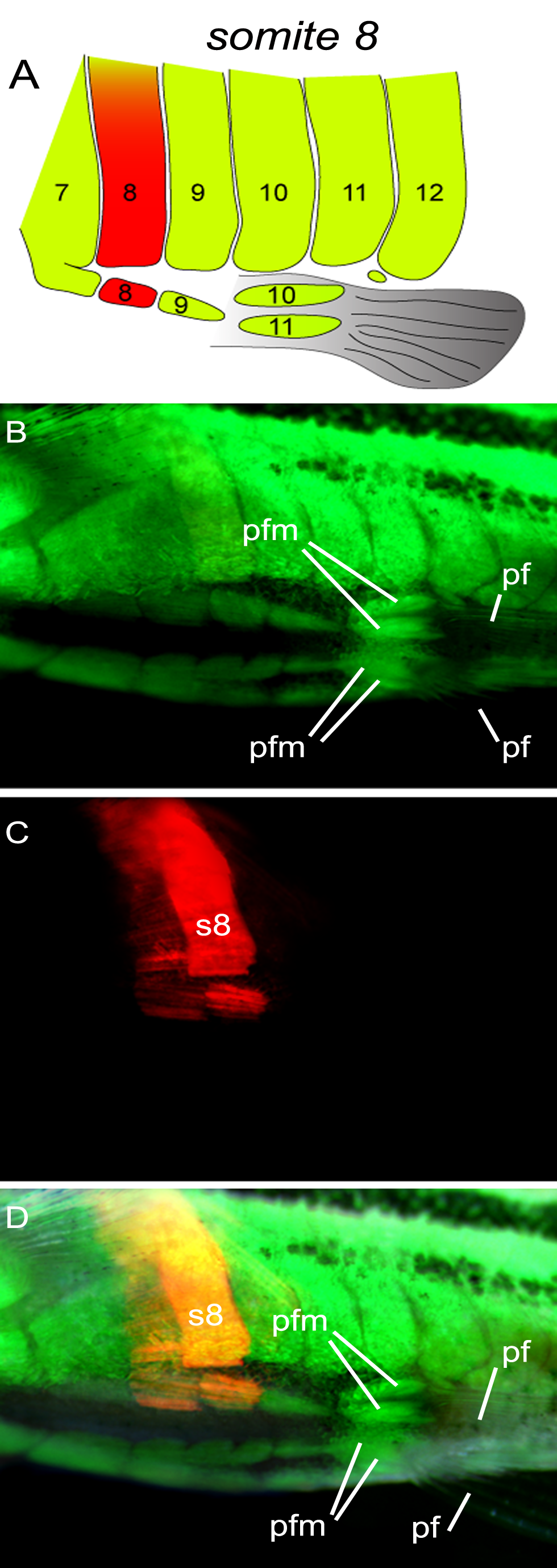

Supplement: Figure S3 — Myotomal extensions derived from somite 8 generate the individual muscle adjacent to the ventral tip of the extending myotomes and anterior to pelvic fin. Pf, pelvic fin; pfm, pelvic fin muscle; rfp, red fluorescent protein; s8, the 8th somite numbered from anterior to posterior. (TIF) [file pbio.1001168.s003.tif]

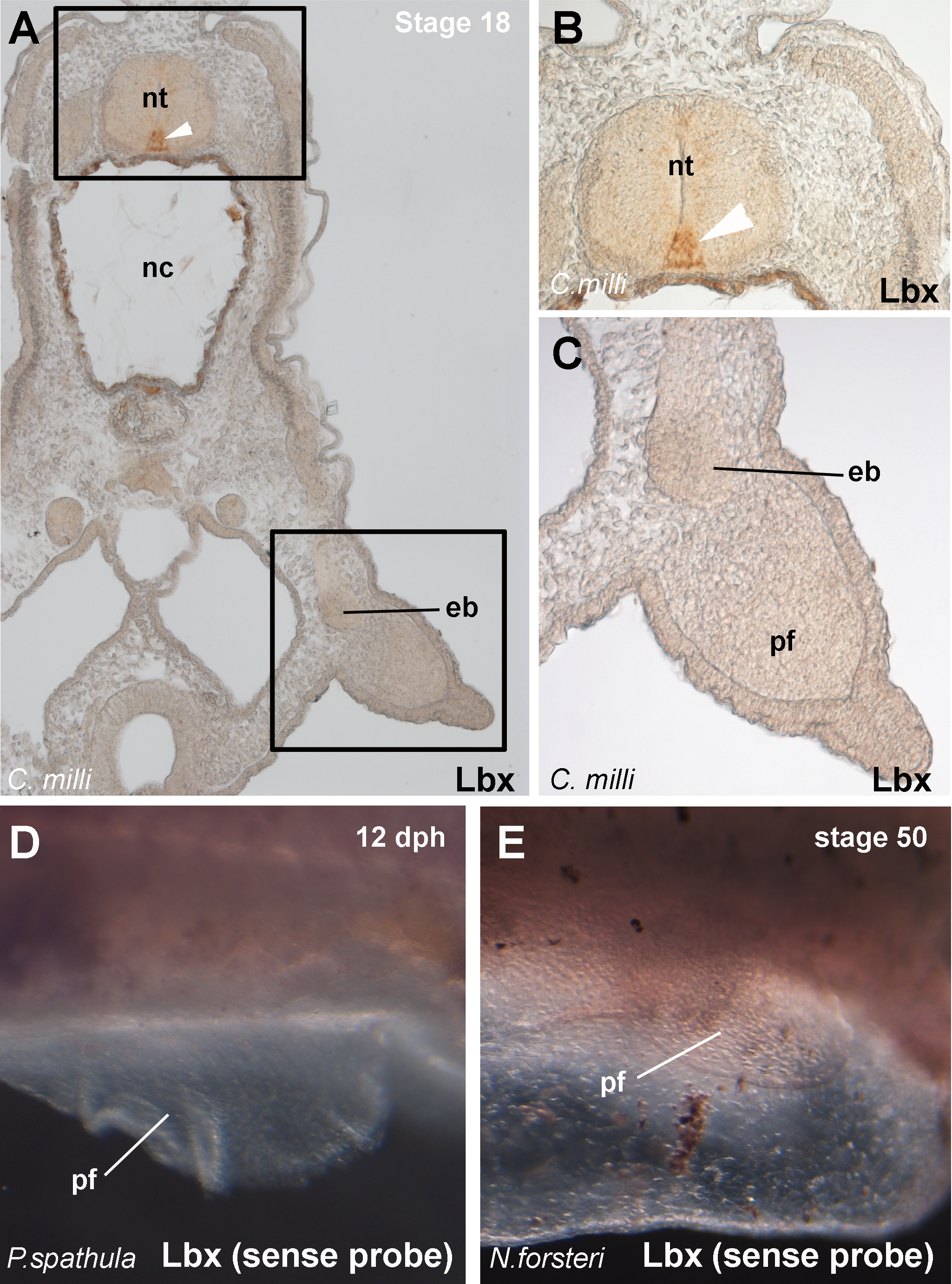

Supplement: Figure S4 — Lbx protein is not detected in the epithelial extension of C. milii. (A) Cross-section at the level of the pelvic fin (pf) of a 28 stage C. milii embryo incubated with an antibody against Lbx1. Expression is detected within the neural tube (nt, arrow head), a known region of expression for Lbx in other species, but is not evident within the epithelial bud (eb) of the myotomal extension. (B) Magnification of the area boxed in (A) showing the expression of Lbx in the neural tube (arrow head). (C) Lbx expression is absent from the epithelial bud of the myotomal extension. (D) Sense probe control for lbx in situ hybridisation on whole mounts of 12 dph paddlefish, the stage utilised in Figure 4J. (E) Sense control for lbx in situ hybridisation on stage 50 lungfish embryos, the stage utilised in Figure 4DD. nc, notochord. (TIF) [file pbio.1001168.s004.tif]
