# Supplementary material for: Development and Evolution of the Muscles of the Pelvic Fin
Source: PLoS Biol. 2011 Oct 4;9(10):e1001168. doi: 10.1371/journal.pbio.1001168 (PMC3186808; doi:10.1371/journal.pbio.1001168)
Supplement: Figure S2 — Identification of lbx sequences in different fish species. lbx1 nucleotide (top) and amino acid (bottom) alignments for human (H. sapiens), lungfish (N. forsteri), zebrafish (D. rerio), paddlefish (P. spathula), and bamboo shark (C. punctatum). Variations between the sequences are shown in grey. (DOC) [file pbio.1001168.s002.doc]

Nucleotide Alignment

human ACGGCCTTCACCAACCACCAGATCTATGAATTGGAAAAGCGCTTTCTATACCAGAAGTAC
lungfish ACGGCCTTTACCAATCACCAGATCTATGAACTGGAGAAGCGATTCCTCTACCAAAAGTAC
zebrafish ACAGCCTTCACCAACCACCAAATCTACGAGCTGGAAAAGAGGTTTTTGTACCAGAAGTAC
paddlefish ACGGCCTTTACCAATCATCAGATCTATGAGCTGGAGAAACGCTTCCTCTATCAGAAATAT
bamboo_shark TCCGCCTTTACCAATCACCAGATTTATGAGCTGGAGAAAAGGTTTTTGCACCAGAAATAC


human CTGTCCCCCGCCGATCGCGACCAAATCGCGCAGCAGCTGGGCCTCACCAACGCGCAAGTC
lungfish TTATCTTCCGCTGATCGGGACCAGATAGCCCAACAGCTGGGTCTCACCAACGCACAGGTT
zebrafish CTCTCACCGGCTGACAGAGACCAGATAGCACAGCAGCTGGGGCTGACCAATGCGCAGGTC
paddlefish CTTTCACCGGCTGACCGGGATCAGATCGCTCAGCAACTCGGCCTGACCAATGCTCAGGTC
bamboo_shark CTGTCGCCCGCAGACAGAGATCAGATCGCTCAGCAACTCGGCCTGACCAATGCTCAGGTC


human ATCACCTGGTTCCAGAATCGGCGCGCTAAGCTCAAGCGGGACCTGGAGGAGAT
lungfish ATCACCTGGTTCCAGAATCGGCGAGCCAAACTCAAGCGGGAACTAGAGGAAAT
zebrafish ATCACCTGGTTCCAGAACCGACGGGCCAAGCTCAAGAGAGACCTTGAAGAGAT
paddlefish ATCACTTGGTTCCAGAACCGCCGGGCCAAACTCAAGCGGGACCTAGAAGAAAT
bamboo_shark ATCACTTGGTTCCAGAACCGCCGGGCCAAACTCAAGCGGGACCTAGAGGAGAA


Amino acid alignment

human TAFTNHQIYELEKRFLYQKYLSPADRDQIAQQLGLTNAQVITWFQNRRAKLKRDLEE
lungfish TAFTNHQIYELEKRFLYQKYLSSADRDQIAQQLGLTNAQVITWFQNRRAKLKRELEE
zebrafish TAFTNHQIYELEKRFLYQKYLSPADRDQIAQQLGLTNAQVITWFQNRRAKLKRDLEE

paddlefish SAFTNHQIYELEKRFLHQKYLSPADRDQIAQQLGLTNAQVITWFQNRRAKLKRDLEE
bamboo_shark TAFTNHQIYELEKRFLYQKYLSPADRDQIAQQLGLTNAQVITWFQNRRAKLKRDLEE

Sup. Figure 2
